# Will It Revert to Pageantry and Burlesque?

**Published:** 1920-01-10

**Authors:** 


### A SHRINKING MINORITY.

### Will it Revert to Pageantry and Burlesque?

THE minority, which for many years has been the chief impediment to the earlier granting of nurse registration by Parliament, has kindly exhibited its true worth by declaring that no nurse who has not read from cover to cover the four volumes of "A History of Nursing," by Professor Adelaide Nutting, R.N., and Miss L. L. Dock, R.N., can be well informed as to the various phases of the development of nursing as a profession or its present position in the various countries of the world. Of this "History of Nursing" the first two volumes have been well done, thanks to the invaluable and powerful aid of Miss Adelaide Nutting, who is so wise a bibliophile as to give her a literary force and status few women possess. These two volumes are history, and will always have a permanent value as such. The last two have failed altogether to attempt, much less to reach, the high standard of the first two volumes, and by all who know the history of nursing in this country have been recognised, and properly defined, if truth has any meaning in this connection, as works of comic humour. Of the four collaborators with Miss Dock in the chapters which deal with the last thirty or forty years of nursing in Great Britain, the photographs of one of these ladies forms the frontispiece, and two of the remainder are similarly immortalised further on. The text speaks of these collaborators in terms of perfervid eulogy, and extols their views, their work and everything that they do, or want to do, in fantastic adulation. It is an exhibition of self-centred and ill-balanced emotionalism, combined with a pitiable breach of good taste, to produce that self-praise which is said to be no great recommendation. Trained British nurses can, therefore, keep their money and need waste no time in perusing the lauding of these four ladies of themselves and each other to the skies, or their belabouring of all those who disagree with them or oppose their projects for the improvement of the status of nurses. This peculiar frame of mind may be of use for the game of mud-slinging; but it does not qualify its possessors for becoming historians, nor make their work useful to the disinterested reader and searcher after truth.

